# Molecular epidemiology of an enterovirus A71 outbreak associated with severe neurological disease, Spain, 2016

**DOI:** 10.2807/1560-7917.ES.2019.24.7.1800089

**Published:** 2019-02-14

**Authors:** Rubén González-Sanz, Didac Casas-Alba, Cristian Launes, Carmen Muñoz-Almagro, María Montserrat Ruiz-García, Mercedes Alonso, María José González-Abad, Gregoria Megías, Nuria Rabella, Margarita del Cuerpo, Mónica Gozalo-Margüello, Alejandro González-Praetorius, Ana Martínez-Sapiña, María José Goyanes-Galán, María Pilar Romero, Cristina Calvo, Andrés Antón, Manuel Imaz, Maitane Aranzamendi, Águeda Hernández-Rodríguez, Antonio Moreno-Docón, Sonia Rey-Cao, Ana Navascués, Almudena Otero, María Cabrerizo

**Affiliations:** 1 Centro Nacional de Microbiología, Instituto de Salud Carlos III, Madrid, Spain; 2 Institut de Recerca Sant Joan de Déu, Barcelona, Spain; 3 Hospital General de Elche, Alicante, Spain; 4 Hospital Infantil Universitario Niño Jesús, Madrid, Spain; 5 Complejo Hospitalario de Burgos, Burgos, Spain; 6 Hospital Santa Creu i Sant Pau, Barcelona, Spain; 7 Hospital Universitario Marqués de Valdecilla, Santander, Spain; 8 Hospital Universitario de Guadalajara, Guadalajara, Spain; 9 Hospital Miguel Servet, Zaragoza, Spain; 10 Hospital Gregorio Marañón, Madrid, Spain; 11 Hospital Universitario La Paz, Fundación IdiPaz, Madrid, Spain; 12 Hospital Universitari Vall d´Hebron, Barcelona, Spain; 13 Hospital de Basurto, Bilbao, Spain; 14 Hospital de Cruces, Bilbao, Spain; 15 Microbiology Service, University Hospital “Germans Trias i Pujol”, Department of Genetics and Microbiology, Autonomous University of Barcelona, Badalona, Spain; 16 Hospital Universitario Virgen de la Arrixaca, Murcia, Spain; 17 Hospital General de Vigo, Vigo, Spain; 18 Complejo Hospitalario de Navarra, Pamplona, Spain; 19 Universitat Internacional de Catalunya, Barcelona, Spain; 20 CIBER de epidemiología y Salud Pública, CIBERESP, Madrid, Spain; 21 Translational Research Network in Paediatric Infectious Diseases (RITIP), IdiPaz, Madrid, Spain

**Keywords:** epidemiology, outbreaks, surveillance, enterovirus, encephalitis

## Abstract

**Introduction:**

Enterovirus A71 (EV-A71) is an emerging pathogen that causes a wide range of disorders including severe neurological manifestations. In the past 20 years, this virus has been associated with large outbreaks of hand, foot and mouth disease with neurological complications in the Asia-Pacific region, while in Europe mainly sporadic cases have been reported. In spring 2016, however, an EV-A71 outbreak associated with severe neurological cases was reported in Catalonia and spread further to other Spanish regions.

**Aim:**

Our objective was to investigate the epidemiology and clinical characteristics of the outbreak.

**Methods:**

We carried out a retrospective study which included 233 EV-A71-positive samples collected during 2016 from hospitalised patients. We analysed the clinical manifestations associated with EV-A71 infections and performed phylogenetic analyses of the 3’-VP1 and 3Dpol regions from all Spanish strains and a set of EV-A71 from other countries.

**Results:**

Most EV-A71 infections were reported in children (mean age: 2.6 years) and the highest incidence was between May and July 2016 (83%). Most isolates (218/233) were classified as subgenogroup C1 and 217 of them were grouped in one cluster phylogenetically related to a new recombinant variant strain associated with severe neurological diseases in Germany and France in 2015 and 2016. Moreover, we found a clear association of EV-A71-C1 infection with severe neurological disorders, brainstem encephalitis being the most commonly reported.

**Conclusion:**

An emerging recombinant variant of EV-A71-C1 was responsible for the large outbreak in 2016 in Spain that was associated with many severe neurological cases.

## Introduction

Enterovirus 71 (EV-A71) is a small, non-enveloped, single-stranded RNA virus that belongs to the species Enterovirus A along with 24 other serotypes within the Enterovirus genus [1]. According to the VP1 protein sequence, EV-A71 is classified into six genogroups (A–F) and a number of subgenogroups (B0–B5, C1–C5) [2]. Although EV-A71 infection is often asymptomatic, it can cause disorders with a wide range of clinical manifestations from non-specific febrile illness, aseptic meningitis and mild mucocutaneous symptoms to severe neurological diseases such as brainstem encephalitis and acute flaccid paralysis (AFP) [3,4]. 

EV-A71 is distributed worldwide. However, the largest outbreaks associated with hand, foot and mouth disease (HFMD) with subsequent neurological and cardiopulmonary complications have been described in the Asia-Pacific region, especially in the past 20 years [2,5-7]. These outbreaks have been connected to the circulation of different subgenogroups (B3, B4, C1, C2 and C4) [8-14]. In Europe, although outbreaks of polio-like disease occurred in Hungary and Bulgaria in the 1970s [15,16], only sporadic cases have been reported from several countries in recent years, mainly caused by the C1 and C2 subgenogroups [2,17]. 

In 2015, a new recombinant EV-A71 variant was identified that affected at least 19 young children in different areas of Germany [18]. This infection was associated with neurological manifestations (cerebral seizures, myoclonia and ataxia) that required hospitalisation. There were no reports of fatal cases or clinical sequelae after hospital discharge. Moreover, a well-documented case, a 2-year-old girl, required hospitalisation and was diagnosed with brainstem encephalitis and cardiopulmonary complications with an outcome of a probable persistent neurological impairment [19]. In addition, 18 cases of severe neurological disease associated with EV-A71 infection, and phylogenetically closely related to the strains described in Germany, were reported in France between May and October 2016. Patients presented with rhombencephalitis, encephalitis or encephalomyelitis, and one fatal case of acute cardiac failure was reported [20]. The same strain was also involved in a sporadic case of encephalitis in Poland during summer 2016 [21]. 

In Spain, EV-A71 was circulating at a very low rate until 2015 [22,23]. In the spring of 2016, however, a large outbreak associated with severe neurological diseases was reported in the region of Catalonia [24-26] and further disseminated to the rest of the country.

In the present study, we investigated the clinical manifestations of EV-A71 infections and the molecular epidemiology and geographical spread of the strains detected in Spain in 2016, comparing them to strains circulating in Spain and other countries in recent years.

## Methods

### Enterovirus surveillance system

The Spanish National Reference Laboratory for Enterovirus (SNRLE) conducts the EV surveillance system at a national level. The system is voluntary and EV-positive clinical samples are received for characterisation from patients of all ages admitted to hospitals throughout the country. Each specimen is sent with information on patient demographics, clinical diagnosis and date of sample collection. For the purposes of this study, more detailed information about the severity of the symptoms, final diagnosis and outcome was subsequently collected from hospitals for patients with neurological disease. EV-positive samples from paediatric patients who were enrolled in a prospective and multicentre project (PI15CIII-0020) were also included in the study after informed consent from parents or legal guardians. This project includes 13 representative hospitals in Spain that perform active surveillance of children with neurological and systemic diseases associated with EV and parechovirus infections. In addition, they provide us with extended clinical information about the patients. The procedure to collect and send samples to the SNRLE does not differ from the routine EV surveillance system.

Clinicians defined the severity of the neurological disorders according to their hospital’s protocols. For this study, a consensus was reached to define severe neurological diseases according to clinical manifestations; it included encephalitis (encephalitis, meningoencephalitis and brainstem encephalitis), AFP/myelitis, and other motor disorders (ataxia, instability, cerebellitis). Mild neurological disease included patients with aseptic meningitis with a good evolution on their own.

### Enterovirus characterisation and phylogenetic analysis

RNA was extracted from clinical samples using the QIAamp Viral RNA Mini Kit (QIAGEN, Germany). EV genotyping was performed using four specific nested RT-PCRs for the species EV-A, B, C and D and further sequencing of the 3’-VP1 region as previously described [27]. In EV-A71-positive samples, the 3Dpol region was amplified and sequenced by specific nested RT-PCR as previously described [28].

We carried out phylogenetic analysis based on 3’-VP1 (359 bp) sequences of EV-71-positive samples. In addition, we performed 3Dpol (738 bp) analysis to confirm possible recombination events [28,29]. Multiple sequence alignments were done in the ClustalW programme. Genetic distances were calculated using the maximum composite likelihood nucleotide distance model, and statistical significance of phylogenies was estimated by bootstrap analysis with 1,000 replicates. Phylogenetic trees were constructed following the neighbour-joining method using MEGA 7.0 software. In the case of 3´VP1, EV-A71 sequences from 2016 were compared with EV-A71 sequences available in GenBank belonging to the C1 and C2 subgenogroups circulating in Spain and other European countries in recent years, along with representative members of the genogroups A, B, C, D, E and F. The coxsackievirus (CV) A16 G-10 prototype strain (CAU05876), a member of Enterovirus A species, was included as an outgroup. For the phylogenetic analysis of 3Dpol, we compared EV-A71 sequences from 2016 with EV-A71 sequences belonging to subgenogroups C1 and C2 as well as sequences of members of the Enterovirus A species with which recombination events normally occur. The EV-D68 Fermon prototype strain (AY426531), a member of Enterovirus D species, was included as an outgroup.

The sequences obtained in this study have been deposited in GenBank under accession numbers MH394906–MH395138 (VP1 sequences) and MH394719–MH394905 (3Dpol sequences).

### Statistical analysis

Significant variations between groups were evaluated using the chi-squared test. A difference with p value < 0.05 was considered to be statistically significant.

## Results

### Virological findings

During 2016, the SNRLE received 1,113 EV-positive samples (mainly CSF, serum, stools and respiratory samples) from 1,029 patients admitted to different Spanish hospitals with clinical pictures of fever without source (FWS) (n = 174; 16.9%), HFMD/exanthema (n = 52; 5.1%), respiratory illnesses (n = 270; 26.2%), the neurological diseases described above (n = 470; 45.7%), and others (n = 63; 6.1%). In the case of 84 patients from whom two different positive clinical samples were received, only the specimen with the oldest collection date was included in the study.

After discarding duplicates, 777 EV were typed, EV-A71 being the most common serotype (n = 233; 30.0%). The following five most frequent serotypes were EV-D68 (n = 148; 19.0%), echovirus (E)-30 (n = 113; 14.5%), E-5 (n = 67; 8.6%), CV-A6 (n = 30; 3.8%) and E-7 (n = 23; 3.0%). Three EV-A71-positive patients were co-infected with EV-D68, one with E-7 and one with E-11.

### Epidemiological data of patients with enterovirus A71

Specimens of EV-A71 were isolated mainly from respiratory (n = 150, 64.4%) and stool samples (n = 71, 30.5%) but were also detected in cerebrospinal fluid (n = 8, 3.4%) and serum (n = 4, 1.7%). Both a respiratory and a stool sample were available in 34 EV-A71 cases. EV-A71 was only successfully genotyped from both samples in 15 of these cases. Patients’ ages ranged from 1 day to 63 years, with a mean of 2.6 years (n = 231 with available information on age; standard deviation (SD) = 5.35; median = 1.86; 95th percentile = 5.9). The male/female sex ratio was 1.5/1. The period from symptom onset to collection date was only provided in 36 cases with a mean of 3.3 days (SD = 1.9; median = 3).

### Phylogenetic analysis of enterovirus-A71

**Figure 1:**
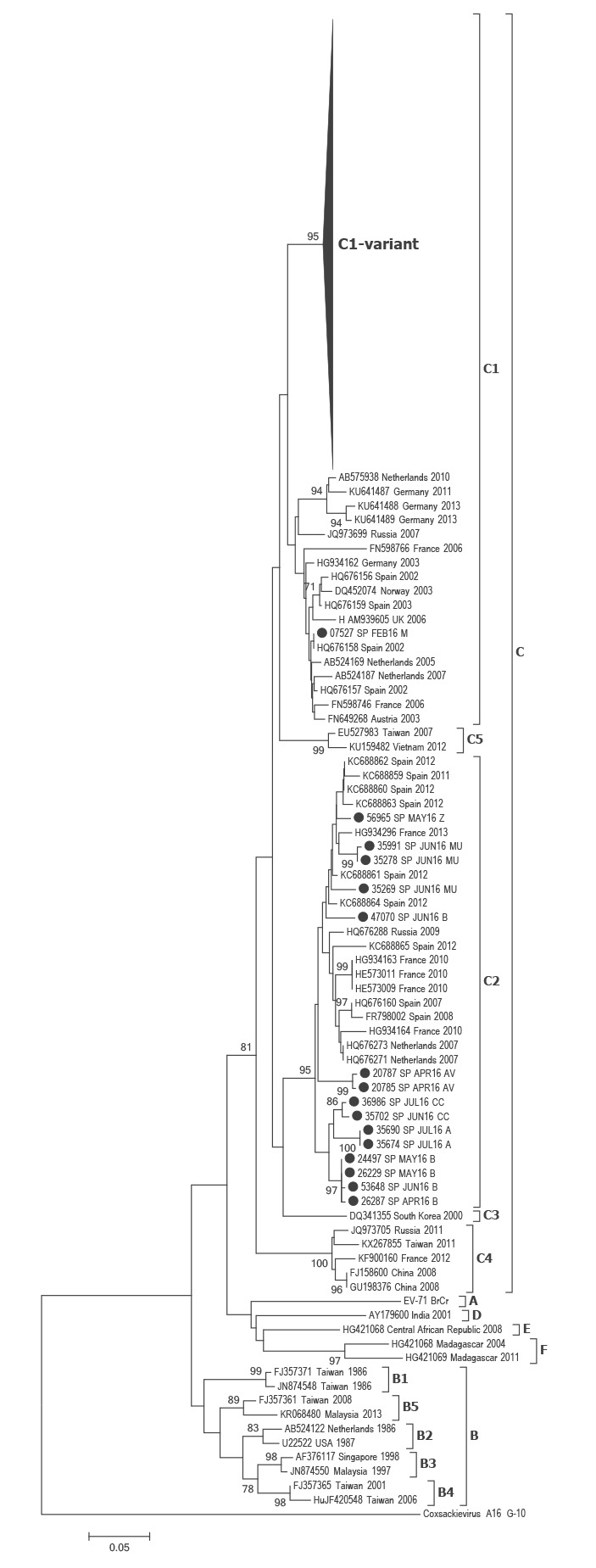
Phylogenetic analysis of 3´-VP1 sequences of enterovirus-A71, Spain, 2016 (n = 233) and representatives of different genogroups worldwide

The VP1 phylogenetic analysis revealed that most of the Spanish sequences from 2016 (218/233) belonged to subgenogroup C1. Of these, 217 formed a separate group within the C1 cluster together with sequences from a new recombinant variant described in Germany in 2015 (bootstrap value > 95%) (Figure 1, Supplementary Figure S1) [18], while one C1 isolate grouped with EV-A71-C1 sequences from previous years. The remaining 15 sequences were grouped within the subgenogroup C2 cluster.

A: Alicante; AV: Ávila; B: Barcelona; CC: Cáceres; EV: enterovirus; M: Madrid; MU: Murcia; Z: Zaragoza.

MEGA 7.0 software was used to construct the neighbour-joining and maximum composite likelihood tree. Bootstrap values > 70% are indicated in the branch nodes. CV-A16 prototype strain G-10 was used as the outgroup. Sequences are labelled with their GenBank accession number, country and year of isolation. Spanish sequences from 2016 are represented by black circles and labelled with identification number, country abbreviation, month and year of isolation and province.

**Figure 2:**
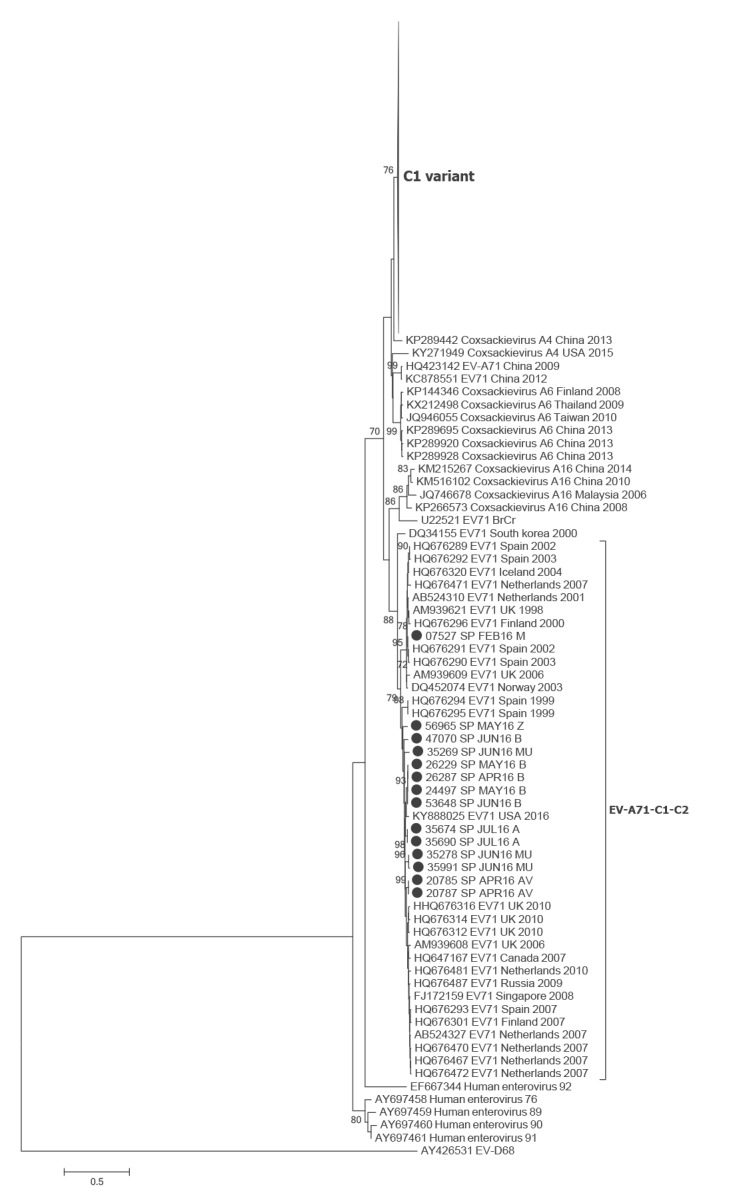
Phylogenetic analysis of 3Dpol sequences of enterovirus-A71, Spain, 2016 (n = 187) and representatives of different members of Enterovirus A species

Because of insufficient sample volume or unsuccessful sequencing, only 187 sequences were available for the 3Dpol phylogenetic analysis. Similarly to the VP1 analysis, most sequences appeared in one differentiated cluster which again included the sequences from Germany in 2015 (bootstrap value > 76%) (Figure 2, Supplementary Figure S2). Interestingly, the same sequences that were grouped in the C2 subgenogroup cluster according to the VP1 sequence, were also grouped separately in an interspersed cluster together with C1 and C2 subgenogroups sequences.

A: Alicante; AV: Ávila; B: Barcelona; EV: enterovirus; M: Madrid; MU: Murcia; Z: Zaragoza.

MEGA 7.0 software was used to construct the neighbour-joining and maximum composite likelihood tree. Bootstrap values > 70% are indicated in the branch nodes. EV-D68 prototype strain Fermon was used as the outgroup. Sequences are labelled with the GenBank accession number, country and year of isolation. Spanish sequences from 2016 are represented by black circles and labelled with country abbreviation, month and year of isolation and province.

### Temporal and geographical distribution of enterovirus-A71

**Figure 3:**
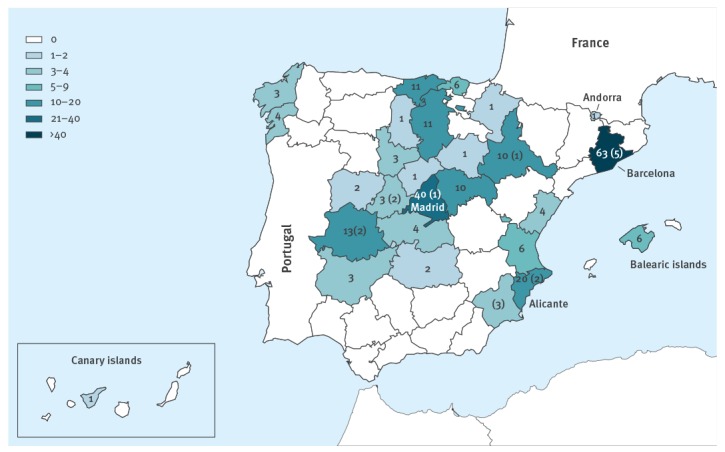
Geographical distribution of enterovirus-A71 cases, Spain, 2016 (n = 233)

**Figure 4:**
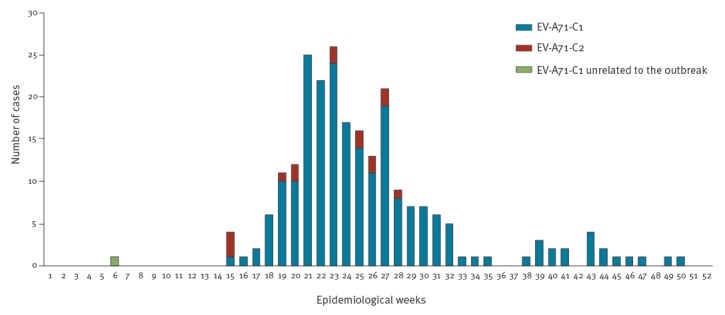
Temporal distribution of enterovirus-A71 cases, by epidemiological week, Spain, 2016 (n = 233)

The total of 233 EV-A71-positive samples were collected from 45 different hospitals in 26 of the 50 Spanish provinces and from Andorra. Barcelona and Madrid, the most populated provinces in Spain, were the two with the highest number of cases (Figure 3). The first cases of the outbreak (subgenogroup C1-variant) were described in Catalonia in April 2016. From May onwards, the number of cases increased, most of them concentrated between May and July (Figure 4), and spread throughout the country. The last two cases were reported in December. A sporadic case associated to subgenogroup C1 but unrelated to the outbreak was reported in February 2016. The cases associated with the subgenogroup C2 were distributed from April to July (Figure 4).

The figure shows the total number of EV-A71 cases reported in each province. EV-A71 cases that were not genotyped as EV-A71-C1 variant are shown in brackets.

Data are represented according to the specimen collection date.

### Clinical presentations of enterovirus-A71 infections

Patients involved in the outbreak (EV-A71-C1-variant; n = 217) were diagnosed with HFMD or herpangina (n = 8; 3.7%), FWS (n = 14; 6.5%), lower respiratory tract infection (n = 17; 7.8%), neurological symptoms (n = 167; 77.0%) and other symptoms (n = 11; 5.1%) (Table). There was a significantly higher proportion of neurological manifestations with EV-A71-C1-variant infection with severe symptomatology (n = 140; 64.5%) compared with those with mild symptomatology (n = 27; 12.4%) (p < 0.0001) (Table). Among the patients infected with EV-A71-C2, five had mild neurological disease, five had severe neurological disease, three had lower respiratory tract infection and two had FWS. The remaining non-outbreak-related EV-A71-C1 case was reported as having FWS (Table).

**Table:** Clinical manifestations among enterovirus-A71 patients, Spain, 2016 (n = 233)

			Number of cases	%	p value	HFMD-associated
**Patients infected with EV-A71-C1 variant (n = 217)**						
Neurological disorders	Severe	Brain stem encephalitis	53	64.5	Not applicable	4
		Meningoencephalitis	47			-
		Encephalitis	18			1
		AFP/myelitis	12			-
		Other motor disorders	10			3
	Mild	Aseptic meningitis	25	12.4	< 0.001	4
		Other	2			-
Lower respiratory tract infection			17	7.8	< 0.001	-
Mucocutaneous symptoms			8	3.7	< 0.001	-
Fever without source			14	6.5	< 0.001	-
Other			11	5.0	< 0.001	-
**Patients infected with EV-A71-C1 or C2 (n = 16)**						
Neurological disorders	Severe	Brain stem encephalitis	2	31.2	Not applicable	-
		Meningoencephalitis	3			-
	Mild	Aseptic meningitis	5	31.2	0.069	1
Lower respiratory tract infection			3	18.8	0.195	-
Fever without source			3	18.8	0.195	-

AFP: acute flaccid paralysis; EV: enterovirus; HFMD: hand, foot and mouth disease.

Significant variations between groups were evaluated by chi-squared test; p values comparing the number of cases with severe neurological disorders with the remaining clinical manifestations are shown.

According to the data provided by hospitals, 22.9% (33/144) of the children with severe neurological diseases required admission to a paediatric intensive care unit and 65.3% of them (94/144) received some treatment (intravenous gamma globulin or/and corticoids). After 90 days of follow-up, 88.2% (n = 127) of them had no significant sequelae.

To confirm the association of EV-A71-C1-variant infection with severity, we analysed the total of severe neurological infections positive for EV during 2016 (n = 190). Meningoencephalitis was the most common clinical manifestation (n = 79; 41.6%) followed by brainstem encephalitis (n = 58; 30.5%), encephalitis (n = 26; 13.7%), AFP/myelitis (n = 17; 8.9%) and other motor disorders (n = 10; 5.3%). EV-A71-C1-variant was responsible for most cases (n = 140; 73.7%) compared with EV-A71-C2 (n = 5, 2.6%) and other EV types that included EV-D68, E-30, CV-B3, E-5, E-7, CV-A6, E-18, CV-A10, CV-A4, CV-B1, E-20, E-6 and E-9 (n = 45, 23.7%) (p < 0.001).

## Discussion

This study presents the clinical and virological characterisation of the EV-A71 strains reported in Spain in 2016. We identified EV-A71 subgenogroup C1 as responsible for a large outbreak that affected young children throughout Spain in 2016 and the clear association of this infection with severe neurological diseases.

Since the outbreaks of polio-like disease in Bulgaria and Hungary in 1975 and 1978 [15,16], the number of EV-A71-associated neurological diseases has been low in Europe compared with the Asia-Pacific region, and most documented EV-A71 infections included sporadic cases of febrile illness, HFMD and meningitis [5,6]. However, several cases of EV-A71 infections associated with severe neurological disorders in children were reported in Germany and France during 2015 and 2016 [18-20]. In Spain, an increasing number of EV-A71 infections was noticed in 2016 compared with previous years. From 2000 to 2015, the SNLRE had identified an average of two isolates of EV-A71 per year with a maximum of seven cases in 2012 [22,23,30].

Regarding specimen types, EV-A71 was mainly detected in either respiratory samples or stools (or both). This is in agreement with several studies that claim that EVs are isolated from CSF only in a minority of patients, whereas recovery from peripheral sites such as throat, stool/rectum and vesicle fluid is more common [3,29]. This finding suggests that in severe neurological disease, respiratory and stool specimens, in addition to the CSF and serum samples, should be submitted for EV testing. 

According to the 3´-VP1 sequence, most EV-A71 sequences in Spain during 2016 (217/233) showed a high similarity with the German strains reported in 2015 and were classified as subgenogroup C1. The fact that according to the 3Dpol sequence our strains also grouped together with the German strains indicates that it is very probably the same recombinant variant, although whole genome sequencing would be necessary to confirm this hypothesis.

We found an association between infection with this recombinant variant and the severity of the neurological manifestations, consistent with the German and French reports [18-20]. In addition, most patients did not present HFMD before developing neurological manifestations (Table), also in agreement with the German and French reports but not with the numerous publications from outbreaks in Asia-Pacific in recent years [31-34]. Several clinical features such as young age or fever have been associated with severe neurological EV-A71 disease [3]. On the other hand, the absence of mucocutaneous ulcers that we saw in patients in our study has not been related to the development of complicated or fatal cases by other studies [3]. Interestingly, most of the 15 EV-A71-C2 cases and the one EV-A71-C1 case unrelated to the outbreak were not associated with severe neurological disease. Since no particular genogroup has been conclusively associated with greater virulence or certain clinical manifestations [5,35] and taking into account that in most cases, host factors play a determining role in the development of the disease, further experiments would need to be performed in order to describe the viral determinants responsible for this new recombinant strain being associated with severe neurological pathology.

The reason why the same recombinant variant caused such a large outbreak in Spain, while only a limited number of cases were reported in Germany and France, remains unknown. Large epidemics have been associated with genogroup replacement [5,36,37]. This would be in agreement with the low circulation of subgenogroup C1 in Spain before 2016 [22,23,30] in contrast to the situation in Germany and France [2,17,38,39]. Low seroprevalence against EV-A71 in a specific area may facilitate the emergence of a strain that causes an outbreak, especially in young children [40]. However, as non-polio EV infection is not a notifiable disease in Spain and asymptomatic strains may have been circulating during recent years, the real number of EV-A71 infections could be underestimated. Unfortunately, no seroprevalence studies are available in Spain to confirm this hypothesis. It is important to highlight that we have identified sporadic cases associated with this EV-A71-C1 variant in 2017 and 2018 (data not shown), indicating that this variant is still circulating in Spain, but with lower incidence. The seasonality of the cases during 2016, including C1 and C2 subgenogroups, was similar to previously described epidemics of EV in Spain [22,23,30]. Moreover, the geographical distribution of the outbreak indicates that it was not restricted to the region of Catalonia, but distributed throughout Spain.

As more severe neurological cases occurred during this outbreak, there was concern among the population, the media and health professionals. Because of this concern, there was constant communication among microbiologists, epidemiologists and clinical paediatricians at national level, in which the National Centre for Microbiology was involved. For this reason, we strongly believe that we studied a large and representative number of cases although we have the limitation of not being able to know with certainty the final number of cases of the outbreak. In addition, it should be noted that we were informed that most severe cases were diagnosed virologically, and only some mild cases were missed [41]. On the other hand, since the Spanish surveillance system is voluntary, there is a risk of having a bias because hospitals are more likely to send samples of severe patients. As mentioned above, 777 EV-positive samples were genotyped at the SNLRE during 2016. Of those, only 190 (25%) belonged to patients with severe neurological disorders. The fact that most samples received in the laboratory during 2016 were from patients with mild symptoms, as well as in previous years, minimises this risk.

Another limitation of our study could be that hospitals may have a bias towards reporting cases in children. Since EV infections are clearly associated with children, it seems that this is a bias than cannot be prevented; however, samples from patients of all ages were received in 2016. Our laboratory has previously described the association of some serotypes with different age groups [23]. Our results suggest an association of infection with EV-A71-C1 variant in young children.

Finally, more Spanish regions could have been affected for which we may not have had data, either because some hospitals did not send samples or because some samples received from reference hospitals, mostly in Barcelona and Madrid, belonged to patients admitted from the surrounding regions. For the purposes of this study, we selected the affected regions according to the hospitals that sent the clinical samples. This is the reason why, for instance, Barcelona is the only province of the region of Catalonia in which the cases were reported.

## Conclusion

In summary, our results show that an emerging EV-A71-C1 strain was responsible for the outbreak in Spain during 2016 and was associated with many severe neurological cases, the largest outbreak in Europe in recent years. This is the first time that this EV-A71-C1 variant has been detected in Spain and it could have its origin in strains from other European countries such as Germany or France. Since poliovirus eradication is a reasonable goal in the short term, surveillance of non-polio EV associated with neurological implications becomes crucial, especially in cases such as EV-A71 infection where no treatments or vaccines are available. In this sense, the recently established European Non-Polio-Enterovirus Network [29] can contribute to standardising methods for EV detection and typing, clarifying the most adequate specimens for testing according to the clinical presentation and, ultimately, monitoring the global circulation of EV types. Our findings highlight the importance of EV surveillance in order to identify new recombinant forms of known EV types and monitor their associated disease burden, their molecular epidemiology and geographical distribution.

## Supplementary Data

492000 Supplement
