# Supplementary material for: Molecular epidemiology of an enterovirus A71 outbreak associated with severe neurological disease, Spain, 2016
Source: Euro Surveill. 2019 Feb 14;24(7):1800089. doi: 10.2807/1560-7917.ES.2019.24.7.1800089 (PMC6381658; doi:10.2807/1560-7917.ES.2019.24.7.1800089)

Supplementary figures. Expanded trees

This supplementary material is hosted by *Eurosurveillance* as supporting information alongside the article "Molecular epidemiology of an enterovirus A71 outbreak associated with severe neurological disease, Spain, 2016." by Rubén Gonzalez-Sanz et al on behalf of the authors who remain responsible for the accuracy and appropriateness of the content. The same standards for ethics, copyright, attributions and permissions as for the article apply. *Eurosurveillance* is not responsible for the maintenance of any links or email addresses provided therein

Supplementary Figure 1. Expanded tree of figure 1.

A-Alicante, AV-Ávila, B-Barcelona, BA-Badajoz, BI-Vizcaya, BU-Burgos, C-La Coruña, CC-Cáceres, CR-Ciudad Real, CS-Castellón, GU-Guadalajara, M-Madrid, MU-Murcia, NA-Navarra, P-Palencia, PM-Palma de Mallorca, PO-Pontevedra, S-Cantabria, SA-Salamanca, SG-Segovia, SO-Soria, TF-Tenerife, TO-Toledo, V-Valencia, VA-Valladolid, Z-Zaragoza.

Isolates are indicated by the GenBank accession number, country and year of isolation. German strains from 2015 are represented by red circles and Spanish strains from 2016 are represented by black circles and indicated by identification number, country abbreviation, month and year of isolation and province.

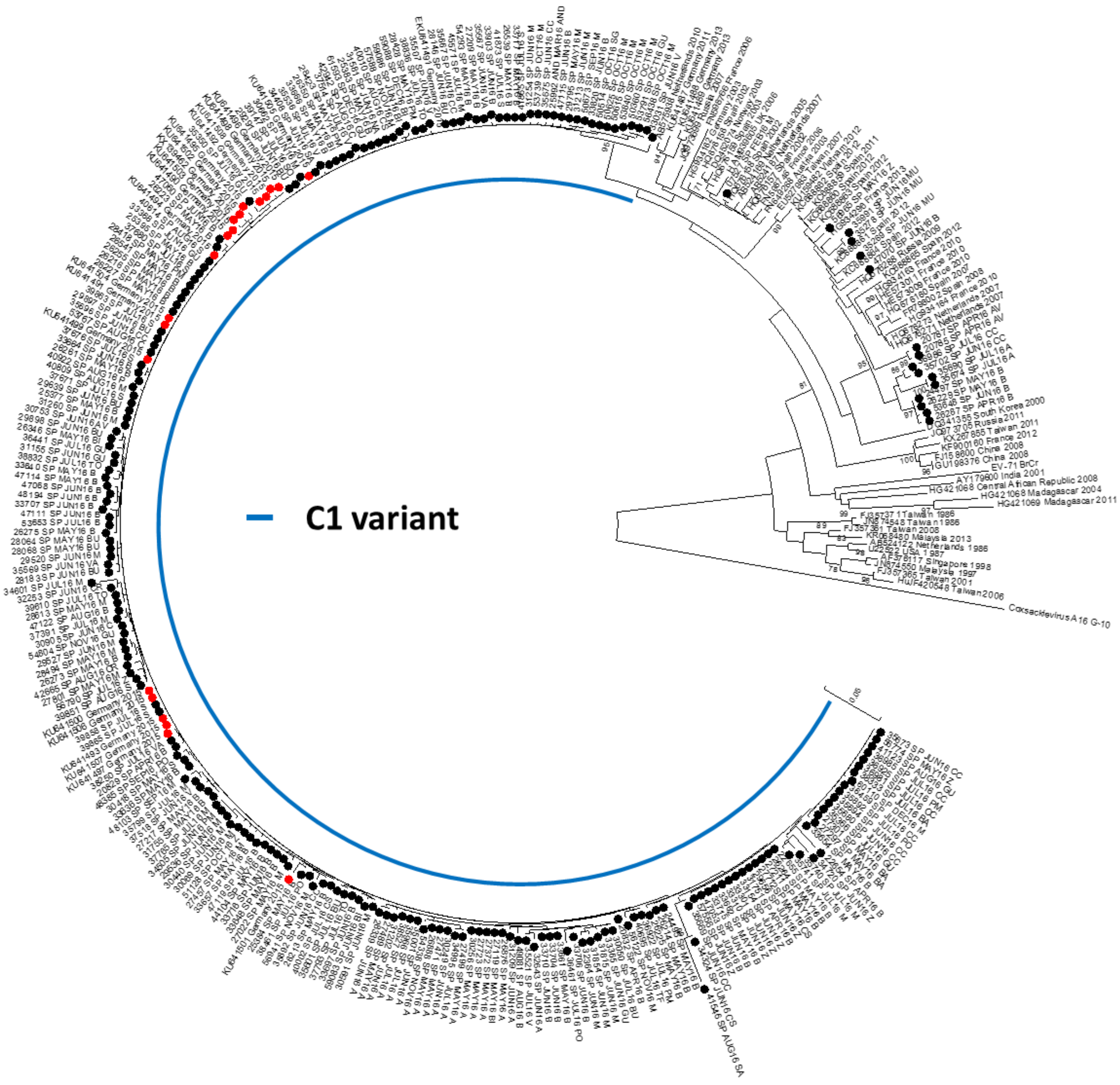

This supplementary material is hosted by *Eurosurveillance* as supporting information alongside the article "Molecular epidemiology of an enterovirus A71 outbreak associated with severe neurological disease, Spain, 2016" by Rubén Gonzalez-Sanz et al on behalf of the authors who remain responsible for the accuracy and appropriateness of the content. The same standards for ethics, copyright, attributions and permissions as for the article apply. *Eurosurveillance* is not responsible for the maintenance of any links or email addresses provided therein.

Suplementary figure 2. Expanded tree of figure 2.

A-Alicante, AV-Ávila, B-Barcelona, BA-Badajoz, BI-Vizcaya, BU-Burgos, C-La Coruña, CC-Cáceres, CR-Ciudad Real, CS-Castellón, GU-Guadalajara, M-Madrid, MU-Murcia, NA-Navarra, P-Palencia, PM-Palma de Mallorca, PO-Pontevedra, S-Cantabria, SA-Salamanca, SG-Segovia, SO-Soria, TF-Tenerife, TO-Toledo, V-Valencia, VA-Valladolid, Z-Zaragoza.

Isolates are indicated by the GenBank accession number, country and year of isolation. German strains from 2015 are represented by red circles and Spanish strains from 2016 are represented by black circles and indicated by identification number, country abbreviation, month and year of isolation and province.

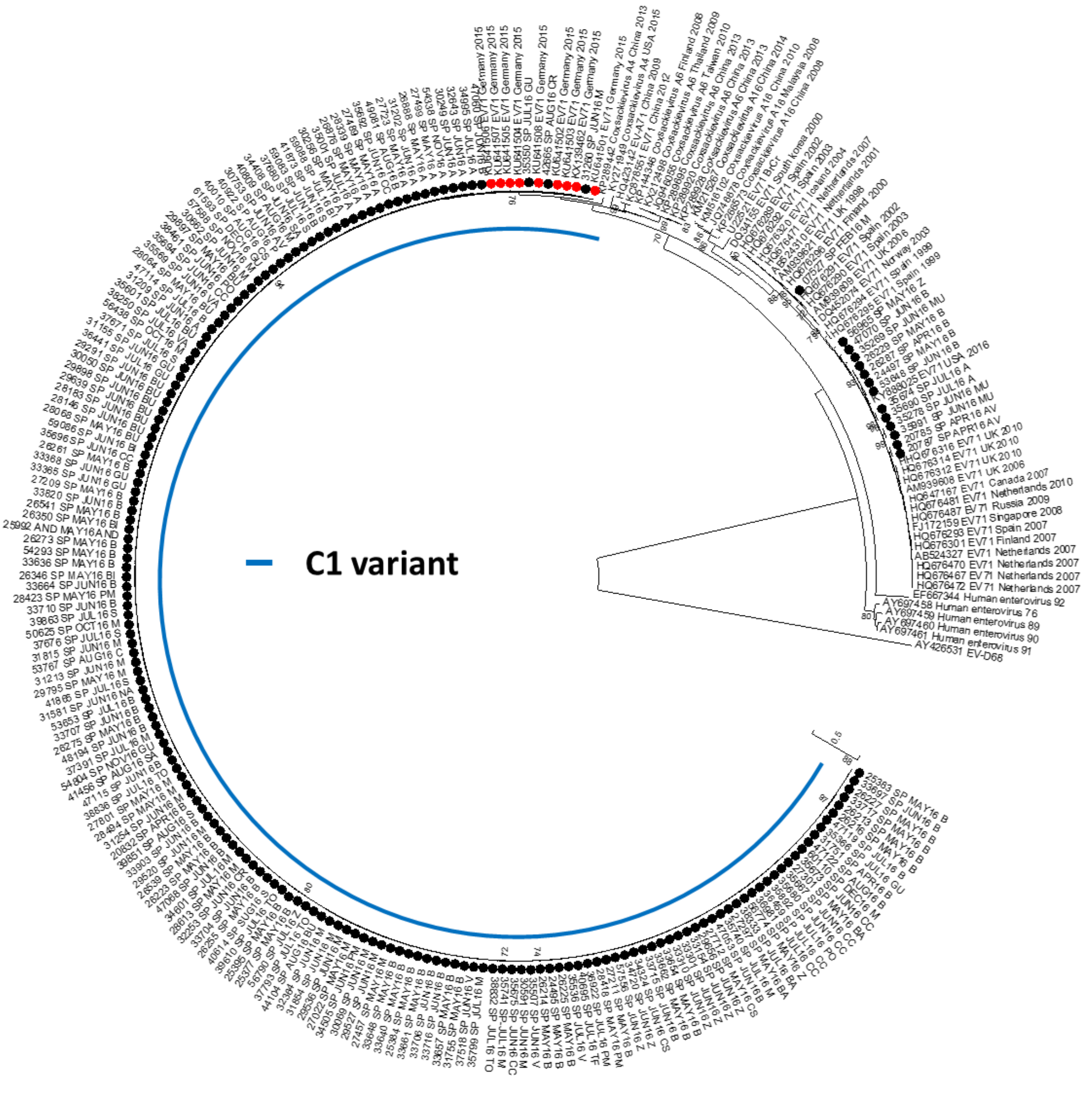

Supplement: Supplement [file 18-00089_Supplement.pdf]
